# High Abundance Proteins Depletion *vs* Low Abundance Proteins Enrichment: Comparison of Methods to Reduce the Plasma Proteome Complexity

**DOI:** 10.1371/journal.pone.0019603

**Published:** 2011-05-04

**Authors:** Renato Millioni, Serena Tolin, Lucia Puricelli, Stefano Sbrignadello, Gian Paolo Fadini, Paolo Tessari, Giorgio Arrigoni

**Affiliations:** 1 Department of Clinical and Experimental Medicine, Division of Metabolism, University of Padua, Padua, Italy; 2 Department of Agricultural Biotechnology, University of Padua, Padua, Italy; 3 VIMM, Venetian Institute of Molecular Medicine, Padua, Italy; 4 Institute of Biomedical Engineering, National Research Council, Padua, Italy; 5 Department of Biological Chemistry, University of Padua, Padua, Italy; Instituto Butantan, Brazil

## Abstract

**Background:**

To date, the complexity of the plasma proteome exceeds the analytical capacity of conventional approaches to isolate lower abundance proteins that may prove to be informative biomarkers. Only complex multistep separation strategies have been able to detect a substantial number of low abundance proteins (<100 ng/ml). The first step of these protocols is generally the depletion of high abundance proteins by the use of immunoaffinity columns or, alternatively, the enrichment of by the use of solid phase hexapeptides ligand libraries.

**Methodology/Principal Findings:**

Here we present a direct comparison of these two approaches. Following either approach, the plasma sample was further fractionated by SCX chromatography and analyzed by RP-LC-MS/MS with a Q-TOF mass spectrometer. The depletion of the 20 most abundant plasma proteins allowed the identification of about 25% more proteins than those detectable following low abundance proteins enrichment. The two datasets are partially overlapping and the identified proteins belong to the same order of magnitude in terms of plasma concentration.

**Conclusions/Significance:**

Our results show that the two approaches give complementary results. However, the enrichment of low abundance proteins has the great advantage of obtaining much larger amount of material that can be used for further fractionations and analyses and emerges also as a cheaper and technically simpler approach. Collectively, these data indicate that the enrichment approach seems more suitable as the first stage of a complex multi-step fractionation protocol.

## Introduction

The human blood is a rich source for biomarker discovery. Plasma is usually preferred over serum for the lower *ex vivo* protein degradation [1], [2].

A comprehensive, systematic characterization of plasma proteome in healthy and diseased states could greatly facilitate the detection of biomarkers for early disease diagnosis, prognosis and therapeutic monitoring. Chances of finding a new biomarker increase with the number of proteins profiled; the most promising source of biomarkers is probably the fraction of low abundant proteins that either leak into the plasma from tissues as a result of disease or play a role as cellular ligands and signal molecules.

However, characterization of the human plasma proteome is a very difficult task: the top ten most abundant plasma proteins account for approximately 90% of the total protein content, while other proteins are present in a very wide dynamic range, spanning more than 10 orders of magnitude in terms of concentration [3].

This last feature, in particular, makes the plasma proteome the most complex human-derived proteome. In fact, current shotgun proteomic technologies are able to detect and identify extremely small amounts of proteins (in the femtomole to attomole range), but have difficulties in detecting and quantifying proteins present at two to three orders of magnitude lower than the most abundant ones. Hence, extensive fractionation is indispensable to reduce the dynamic range and enhance the coverage of the plasma proteome. The recent review of Hoffman et al. [4] describes the increasingly complex approaches that have been developed over time, starting with single-step protocols (leading to the identification of ∼100 proteins), to more complex 4-step protocols (where over 2000 proteins could be identified). This trend is confirmed by works published after 2007 [5]–[8].

Since the analysis of plasma proteome necessarily requires a multidimensional approach, it is particularly important to optimize each step, in order to get the best results.

In almost all plasma proteome studies, the first step is immunodepletion of high abundance proteins (HAPs), a step that is necessary for detection of low abundance proteins (LAPs).

Several studies on the efficiency, reproducibility and non-specific binding of different depletion products have been already published [6], [9]–[21]. The majority of these studies, however, only assessed HSA or HSA and IgG removal [10], [11], [14], [19], [21].

During the last years, there has been a gradual development of several multiple affinity removal columns for the simultaneous depletion of even more HAPs, able to retain 7 (e.g. the MARS Hu-7 kit by Agilent Technologies), 14 (e.g. the Seppro IgY14 kit by Sigma Aldrich or the MARS Hu-14 kit by Agilent Technologies) and 20 HAPs (e.g. the ProteoPrep20 by Sigma).

An alternative and innovative strategy to isolate LAPs is based on the treatment of complex protein samples with a large, highly diverse library of hexapeptides bound to a chromatographic support (ProteoMiner technology, BioRad). In theory, each unique hexapeptide binds to a unique protein recognition site. Since HAPs saturate their ligands, exceeding proteins are washed out during the procedure. In contrast, LAPs are concentrated on their specific ligands, thereby decreasing the dynamic range of proteins in the sample [22].

The literature is actually limited in comparing these two major approaches: to the best of our knowledge, there are currently only five published papers comparing HAPs depletion and LAPs enrichment [8], [23]–[26], and none of them included the ProteoPrep20 which immunocaptures the highest number of HAPs and therefore should be considered the more efficient currently available depletion system. From the literature, it appears that depletion of only HSA and IgG is less efficient compared to the use of peptide ligand affinity beads [25]. The literature is inconsistent and controversial in the comparison between more complex multi-depletion systems and LAPs enrichment approach. In fact, some authors state that removal of up to 12 [8] and 14 [23], [24] HAPs gives a similar performance as LAPs enrichment, while other authors [26] showed that MARS Hu-7 depletion kit performance surpassed that of ProteoMiner.

Many of the above mentioned studies concerning the comparison between different depletion systems or the comparison between depletion and enrichment methods were conducted using 2-DE. The evaluation criterion was based on the number of visualized spots in the gel, without giving information of protein identities.

This way of comparing performances is misleading, considering that the high abundant proteins in the plasma are also present in many different isoforms that appear as different spots in a 2D gel. Therefore, a higher number of spots visible on a gel could be indicative of an incomplete or partial depletion rather then of a more efficient depletion. Conversely, it is essential to identify the proteins and classify them according to protein families in order to compare the real capacity of the depletion or the enrichment methods, to remove the highly abundant proteins or enrich the low abundant ones. For these reasons, in order to compare depletion and enrichment methods, we have decided to use a gel-free approach.

The aim of this study was to determine which method between HAPs depletion and LAPs enrichment provides the best overall results in terms of number of identified proteins, protein coverage and enhanced sensitivity limit. In particular, for the first time, we compared the results obtained using ProteoMiner to those obtained using ProteoPrep20, which is currently the deepest depletion spin column kit commercially available.

## Materials and Methods

All chemicals used in this study were of sequencing grade and were purchased from Sigma (St. Louis, MO, USA) unless otherwise specified.

### Plasma sample

The protocol was approved by the Ethics Committee of the Medical Faculty at the University of Padova, Italy, and was performed according to the Helsinki Declaration (1983 revision). The human blood sample was harvested in EDTA collection tubes by a healthy donor who provided a written informed consent. After centrifugation at 1500 RCF for 10 min, plasma was separated from blood cells and a cocktail of protease inhibitors (AEBSF, Sigma-Aldrich, St. Louis, USA) was added. Plasma was stored at −20°C until use.

### HAP depletion procedure

#### ProteoPrep20 depletion

The ProteoPrep20 immunodepletion spin column technology (PROT20S-1KIT, Sigma-Aldrich, MO, USA) employs a mixture of antibodies against the following 20 human plasma HAPs: albumin, transferrin, alpha-1 acid glycoprotein, Complement (C1q, C3, C4), Ig (G, A, M, D), fibrinogen, ceruloplasmin, alpha-2-macroglobulin, alpha-1-antitrypsin, apolipoprotein (A-1, A-II, B), plasminogen, haptoglobin, prealbumin. According to the manufacturer instructions, 8 µl of plasma sample were diluted to 100 µl with PBS, filtered (0.2 µm) through a Corning Spin-X Centrifuge Tube Filter, added to the immunoaffinity spin column (previously equilibrated in PBS) and incubated at room temperature for 20 min. The column was then centrifuged at 1500 RCF for 1 min and the flow-through (depleted plasma) was collected in a clean tube. The remaining unbound proteins were further washed from the spin column by adding 100 µl of PBS and collected in the same tube by centrifugation. This washing step was repeated twice. Sample was concentrated with an Ultrafree-MC microcentrifuge filters to a final volume of 125 µl. The column was finally regenerated by removing the bound proteins with 2 ml of Elution Solution (0.1 M Glycine-HCl, pH 2.5, and TWEEN 20) as specified by the manufacturer. The column was stored at 5°C in 5 ml of Equilibration Buffer (phosphate buffered saline) with the addition of 10 µl of ProteoPrep Preservative Concentrate.

### Multi-step depletion approach: combination of ProteoExtract and ProteoPrep20 depletion

HSA and IgG depletion using the ProteoExtract kit (1^st^ passage) (ProteoExtract Albumin/IgG removal kit, catalog #122642, Calbiochem, EMD Biosciences, CA, USA) was performed as previously described [27]. Briefly, 60 µl of plasma were diluted in 400 µl of sodium phosphate buffer 0.1 M pH 7.5, applied to the affinity column, to accomplish the specific binding of HSA and IgG and the eluate was collected together with 1200 µl of sodium phosphate buffer 0.1 M pH 7.5, used to wash the column. The HSA- and IgG-free sample was concentrated to 300 µl with Vivaspin 500 centrifugal concentrators and further depleted (100 µl at a time) using the ProteoPrep20 column as described above, loading 100 µl at a time (2^nd^ passage). The 3 fractions eluted from the ProteoPrep20 column were pooled and concentrated with Vivaspin 500 down to 100 µl. Finally, these 100 µl were depleted one more time with ProteoPrep20 (3^rd^ passage).

#### LAP enrichment procedure

LAPs enrichment was performed using the ProteoMiner technology (ProteoMiner Introductory kit, catalog #163-3001, BioRad, CA, USA), which is based on a combinatorial library of hexapeptides bound to a chromatographic support. According to the manufacturer instructions, storage solution was removed by centrifugation at 1000 RCF for 2 min and the bead column was washed first with deionized water and then with 25 mM phosphate buffer, pH 7.4. 1 ml of plasma was added to the column and incubated at room temperature for 2 h. Unbound material was removed by centrifugation and the column was washed three times with 25 mM phosphate buffer, pH 7.4 and once with deionized water. Bound proteins were incubated for 15 min and sequentially eluted with with 100 µl of elution buffer 1 (7 M urea, 2 M thiourea and 3% CHAPS) and 100 µl of elution buffer 2 (9 M urea in 50 mM acetic acid or citric acid, pH 3.3, 2% CHAPS). Finally, the two eluted samples were pooled and analysed.

#### Protein precipitation, quantification and tryptic digestion

Proteins obtained by the different fractionation methods were precipitated in four volumes of cold acetone (100%) overnight at −20°C. Samples were then centrifuged at 14000 RCF for 10 min and pellets were dissolved in 100 µl of 20 mM ammonium bicarbonate. Protein concentration was determined by a Lowry assay [28]. Proteins were incubated overnight at 37°C with sequencing grade trypsin (Promega, Madison, WI, USA) and with an enzyme to substrate ratio of 1∶20 (w/w).

#### Strong Cation Exchange peptide fractionation

After tryptic digestion, SCX fractionation was performed using a cation exchange cartridge (AB Sciex, Toronto, Canada). Samples were diluted to 500 µl in equilibration buffer (5 mM KH_2_PO_4_, 25% acetonitrile, pH 3), adjusting the pH with 1 M H_3_PO_4_. Peptides were loaded onto the cartridge at 50 µl/min and extensive washing was performed with 1 ml of equilibration buffer. Peptides were fractionated and stepwise eluted using each time 500 µl of elution buffer (5 mM KH_2_PO_4_, 25% acetonitrile, pH 3, with the addition of 50, 100, 150, 200 and 350 mM KCl). Peptide fractions were dried under vacuum, resuspended in 1 ml of 0.1% formic acid and desalted with C18 cartridges (Strata, Phenomenex) according to the manufacturer instructions. Desalted samples were finally dried under vacuum, dissolved in 20 µl of 0.1% formic acid and analyzed by LC-MS/MS.

#### Reversed-phase LC-MS/MS analyses

Peptides obtained by SCX fractionation were analyzed by LC-MS/MS using a 6520 Q-TOF mass spectrometer coupled online with a 1200 series HPLC system through a Chip Cube Interface (Agilent Technologies, CA, USA). Five µl of each sample were loaded onto a C18 large capacity chip-column that integrates a 160 nl capacity trap-column, a RP column (75 µm×150 mm), connection capillaries, and nanospray emitter. Peptides were separated with a linear gradient of 0–50% of solvent B in 50 min at a flow rate of 0.5 µl/min. Solvent A was water/formic acid 0.1%, while solvent B was acetonitrile/formic acid 0.1%. Mass spectra were acquired in a data dependent mode: MS/MS spectra of the 3 most intense ions were acquired for each MS scan in the range of 350–2400 Da. Scan speed was set to 4 MS spectra/sec and 3 MS/MS spectra/sec. Capillary voltage was set to 1750 V and drying gas to 5 l/sec. Raw data files were converted into Mascot Generic Format (MGF) files with MassHunter Qualitative Analysis Software (Agilent Technologies) and analyzed using Proteome Discoverer Software (version 1.2, ThermoFisher Scientific, CA, USA) as described below. Starting from the confidently identified peptides, an excluding list was made for each sample and the LC-MS/MS analysis was repeated using the same chromatographic conditions and the same acquisition method. The new raw data files were then converted into MGF format and merged with the previous acquired files in order to obtain a single MGF file for each sample.

#### Data analysis

The MGF files were analyzed using Proteome Discoverer 1.2 (Thermo Fisher Scientific). The software was connected to a Mascot Search Engine server version 2.2.4 (Matrix Science, London, UK) and to a Sequest Search Engine version 28.0 (ThermoFisher Scientific). Spectra were searched against the IPI Human database (version 24 February 2010, 86719 entries) with the following parameters: enzyme specificity was set to Trypsin with up to 2 missed cleavages, peptide and fragment tolerance were set to 10 ppm and 0.05 Da respectively. Oxidation of Methionine was selected as variable modification. False Discovery Rates (FDR) of 0.5% and 0.1% were calculated by Proteome Discoverer based on the search against the corresponding randomized database. Before the search, data were filtered to exclude MS/MS spectra containing less than 5 peaks and with a total ion count lower than 50. MGF files were searched against Mascot only, or against Mascot and Sequest followed by merging the results into a single list of peptides and proteins. For Sequest analysis, peptides were validated after meeting the following criteria: the cross-correlation score had to be ≥1.9 for +2 tryptic peptides, ≥2.5 for +3 and +4 tryptic peptides for medium confidence, while for high confidence identifications XCorr should be be ≥2 for +2 tryptic peptides and ≥2.8 for +3 and +4 tryptic peptides. Identified peptides were classified as high (99%) and medium (95%) confidence, according to the corresponding FDR.

Proteins were considered as positive hits if at least two peptides with medium confidence were identified per protein or if one peptide was identified with high confidence. Results are reported as single identified proteins or as protein groups, i.e. the minimum set of protein sequences that adequately accounts for all observed peptides.

## Results

**Figure 1:**
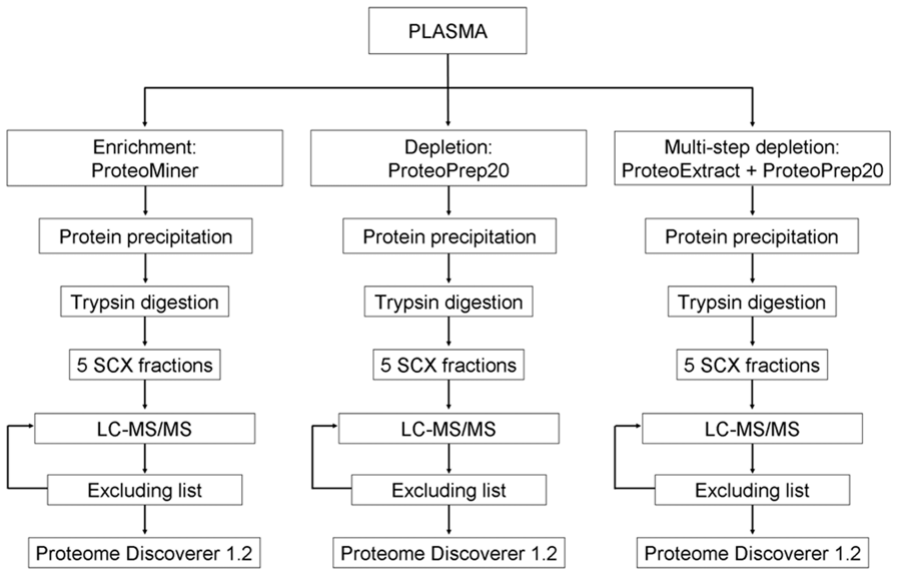
Experimental workflow.

The experimental workflow is presented in Figure 1. We compared the results obtained with analyses of a plasma proteome derived from an immunoaffinity depletion of 20 highly abundance proteins and the enrichment of low abundance proteins by chemical hexapeptide libraries. Moreover, a multi-step depletion was also performed, as described in the methods section. For each fractionation approach, the number of peptides, proteins, and protein groups identified by 1 (95% and 99% confidence), 2, and >2 (95% confidence) peptides are reported in Table 1. Our analyses led to an average identification of a few hundred proteins, a result that is in line with those published in similar studies [8], [23]. However, the number of identified proteins varies significantly depending on the criteria chosen to consider the identification as a positive hit (% confidence and minimum number of peptides per protein). For each experimental approach, the complete list of identified proteins is reported in Table S1 of supplemental data.

**Table 1:** Number of peptides, proteins, and protein groups identified by 1 (95% and 99% confidence), 2, and >2 (95% confidence) peptides for each fractionation approach.

Fractionation approach	n° of peptides (95% conf)	n° of proteins (protein groups) identified			
		1 peptide (95% conf)	1 peptide (99% conf)	2 peptides (95% conf)	>2 peptides (95% conf)
ProteoMiner	2370	484 (195)	318 (139)	197 (90)	143 (66)
ProteoPrep20	3966	557 (271)	334 (186)	226 (130)	157 (92)
ProteoExtract+ProteoPrep20	3670	644 (239)	429 (163)	271 (123)	195 (92)

Considering the complexity of the dataset and the high variability due to the different criteria, we decided to focus the discussion on the number and type of protein groups identified with 99% confidence and one peptide per protein [29].

Using these criteria, the merging of all sets of data allowed to identify a total of 279 protein groups (Table S1). The average sequence coverage calculated for the three methods gave very similar results.

## Discussion

The most straightforward result of this study, as can be deduced from Table 1, is the lower efficacy of the ProteoMiner approach in terms of total number of proteins identified, while the immunodepletion and the multi-step depletion approaches led to a similar number of positive identifications. The same result was found for the total number of peptides. What is striking to notice is the number of proteins or protein groups that are identified with only one significant peptide. In average, for about 30% of the proteins, only one specific peptide was sequenced (compare columns 4 and 5 in Table 1). These results are in line with what has been reported in other studies, where the contribution of single peptide identifications is also quite large [30], [31].

Although the enrichment method led to a lower number of protein identifications, the protocol is much simpler and faster compared to the depletion approach and requires less sample manipulations. This advantage of the enrichment over the depletion protocol is evident when considering the number of contaminant proteins (typically keratins) that were identified. The keratin contamination is almost negligible in the plasma sample treated with ProteoMiner, while for the sample depleted with ProteoPrep20, keratin peptides account for almost 10% of the total number. This can be ascribed to the very laborious procedure of plasma depletion that requires a heavy and time-consuming handling of the sample. Obviously, to allow such a comparison, the operator, the quality of reagents, and the technical precautions were identical for both approaches and the 2 procedures were conducted in parallel.

Moreover, the protocol suggested by the supplier of ProteoPrep20 indicates, as an optional step, the precipitation of proteins prior to trypsin digestion and MS analysis (i.e. after the depletion procedure). We clearly verified that such a procedure cannot be considered as optional because of the high amount of polymeric compounds released by the depletion column that strongly interferes with the MS analysis by suppressing peptide ionization. We could demonstrate that plastic contaminants are released into the sample not only from the depletion column itself, but also when filtering the plasma at the preliminary step and when concentrating the final depleted sample with the provided concentrator (see methods section). An example of base peak chromatograms obtained after each step of the depletion protocol applied to an ultra-pure water sample and a characteristic MS spectrum of the contaminants are shown in supplementary Figure S1.

Table 2 reports the number of peptides, proteins and protein groups that were identified with at least one peptide and 99% confidence with Mascot, Sequest and the combination of the two. Our results show that Sequest clearly outperforms Mascot in terms of number of peptides identified. A manual screening of the identified peptides suggests that Sequest is able to identify more modified peptides (Met-Ox) and more often identifies the same peptides with different charge states, while Mascot generally fails to do so. However, by looking at the MS/MS spectra, it is possible to deduce that Sequest is less stringent in terms of spectral quality. A partial overlapping of the results could be observed, but an in-depth statistical analysis should be performed in order to characterize the common features of the peptides that are better identified by one or the other of the search engines. The combination of the two types of software does not increase significantly the total number of proteins identified, but it positively affects the average sequence coverage.

**Table 2:** Number of peptides, proteins and protein groups that were positively identified with at least 1 peptide and 99% confidence with Mascot, Sequest and the combination of the two search engines.

Fractionation approach	MASCOT			SEQUEST			MASCOT+SEQUEST		
	Peptides	Proteins	Protein Groups	Peptides	Proteins	Protein Groups	Peptides	Proteins	Protein Groups
ProteoMiner	363	147	72	1551	305	136	1914	318	139
ProteoPrep20	897	232	131	1798	323	183	2695	334	186
ProteoExtract+ProteoPrep20	861	258	112	1668	418	161	2529	429	163

The limited number of proteins identified in this study, despite a multi-step approach (enrichment/depletion, SCX, RP-LC-MS/MS), highlights the difficulty of analysing the plasma proteome. The merging of all sets of data allowed the identification of a total of 279 unique protein groups with a 99% confidence. However, our aim was not to develop a protocol for the identification of the maximum number of proteins in plasma, but rather to evaluate which of the two methods, between HAPs depletion and LAPs enrichment, is more suitable as the first step for a plasma proteomic analysis.

Despite the different number of identifications, all the fractionation approaches primarily led to the detection of proteins related to acute phase reaction, and complement and coagulation, including proteins which can be classified as high- (1–100 mg/ml) and mid- (0.1–1 mg/ml) abundance plasma proteins.

**Figure 2:**
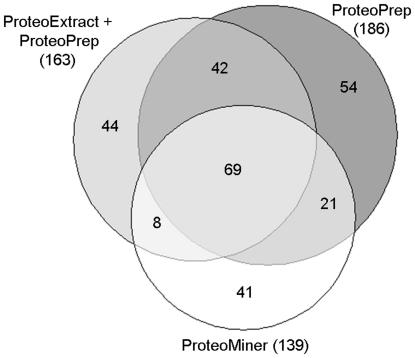
Venn diagram. The diagram shows the overlap of protein groups identified after the different plasma fractionation approaches.

To show the overlap among the fractionation methods, we report in Figure 2 a Venn diagram of the protein groups identified with 99% confidence and one peptide per protein. From this diagram it is clear that the three experimental protocols are complementary: only 69 protein groups are common to all the approaches, which represent only 37, 42, and 50% of all groups associated to ProteoPrep20, ProteoExtract+ProteoPrep20, and ProteoMiner respectively. By looking at the list of proteins identified and the Venn diagram, we conclude that the great majority of proteins, regardless the fact that they are identified with one or more methods, belong to the above mentioned categories. Therefore, all methods yielded similar performance in terms of concentration range of identified proteins.

These data may suggest that the depletion and the enrichment methods we have compared exhibit a similar performance and lead to partially overlapping results. However, there are several other aspects to be taken into account.

The aim of a plasma proteome analysis is to study proteins with a concentration under 100 ng/ml, so other steps will necessarily follow the first to improve sensitivity of the analysis. In this regard, the use of ProteoPrep20, as a first-line fractionation step, does not seem very practical. The major limit of this depletion kit is its reduced plasma loading capacity of only 8 µl. In fact, the amount of proteins obtainable after ProteoPrep20 depletion is of only 17 µg, which can be a limit for performing further fractionations. Since the ProteoPrep20 column is recyclable up to 100 times, one possibility could be to repeat the depletion many times and pool the depleted fractions, with the inconvenience of time consuming and extensive manipulation (with an increasing risk of introducing errors and contaminations, as already discussed above). For the sake of completeness it is important to report here that ProteoPrep20 is also commercially available in form of a LC column that has a much larger loading capacity than the small columns provided with the immunodepetion kit used in this study. Such LC columns can be loaded with up to 100 µl of plasma, are reusable up to 100 times and are probably not subjected to the release of polymeric compounds if properly conditioned. However, ProteoPrep20 LC column has an estimated cost of 12,000 € and therefore it is not easily affordable.

Another point to consider is the following: after using the multi-affinity system, among the unbound identified proteins, we detected with high coverage almost all the 20 proteins that should have been depleted (Table S1). This undesired effect probably depends on the fact that the column-bound antibodies although polyclonal, do not recognize all isoforms and fragments of the 20 HAPs, or because the quantity of the HAPs saturates and exceeds the column binding capacity (even when not overloaded). In an attempt to improve the depletion approach, we decided to make a first depletion with the ProteoExtract columns which are able to retain 70% of HSA and IgG, starting from 60 µl of plasma. Since these columns use an affinity resin and not antibodies, we thought they could be used in a complementary way with ProteoPrep20 to eliminate, at least, the largest possible amount of HSA and IgG. Moreover, thanks to this first depletion, we aimed at loading into the ProteoPrep20 column a larger amount of LAPs compared to the undepleted plasma. The HSA and IgG depleted sample was divided into several aliquots which were then depleted by ProteoPrep20 column one by one. Thereafter, these eluates were pooled, concentrated and depleted again with ProteoPrep20. Even this multi-step depletion approach did not allow the complete removal of high abundance proteins, although the number of peptides belonging to these proteins was reduced. Moreover, despite the multi-depletion approach, the number of identified peptides and proteins, the average sequence coverage, and the sensitivity limit of the analysis were similar compared to those obtained after a single depletion with ProteoPrep20 (see Table 1).

A further attempt to improve the analyses, by analyzing again the same samples using the same parameters and chromatographic conditions, but applying an exclusion list (see Materials and Methods, section 2.6) resulted only in the increase of the percent coverage of some proteins already identified.

This partly confirms that, to dig deeper into the plasma proteome, the most appropriate strategy of analysis is to include additional steps and separate proteins on the basis of many different criteria, such as the specific capture of glycol- and cysteinyl-peptides [32], [33].

While ProteoPrep20 kit was developed specifically for the plasma analysis, the ProteoMiner approach, even if it is a relatively recently developed technology, has already been applied to the study of proteome from urine [34], serum [35], [36], platelets [37], and red blood cells [38]. This novel fractionation method employs a large, highly diverse bead-based library of combinatorial peptide ligands, which simultaneously reduces HAPs and enriches LAPs.

The recovery of proteins after LAPs enrichment is approximately 3%, which is the same recovery obtained after the ProteoPrep20 depletion. In terms of amount of proteins, however, ProteoMiner allows obtaining a quantity 150 times greater and this depends on the high capacities of the column (1 ml). Although LAPs enrichment led to the identification of fewer proteins (about 25%) with respect to ProteoPrep20 depletion, we must take into account the great advantage of obtaining sufficient material that can still be subjected to further analysis. The reduced plasma load capacity of immunodepletion column and the subsequent necessity to reuse them many times is a common feature of all the commercially available depletion kits [39].

Most authors who have recently conducted similar studies using other protocols and kits, but always comparing LAPs enrichment *vs* HAPs depletion as the first step of their protocol, have concluded that the two methods are complementary, as their records indicate that these methods allow to obtain similar and only partially overlapping results [8], [23], [24]. From these statements a very interesting and stimulating debate may emerge. We speculate that the idea of combining the two techniques in a complementary way is not feasible. We retain the view that, for practical aspects, the LAPs enrichment approach is an appealing fractionation technique. Indeed, given the huge amount of work that a proteomic analysis of plasma requires, it is preferable to develop a single orthogonal protocol consisting of several steps to detect the proteins in the 100 ng/ml range, rather than create and merge results from multiple parallel analyses, because each single analysis might not reach the desired sensitivity level.

Despite the continuous development of columns able to deplete more and more HAPs simultaneously with the aim to reach the low-abundance plasma protein range (<100 ng/ml), the approach of raising the number of antibodies may become a prohibitively expensive (and never-ending) strategy, with a parallel increase of nonspecific binding, which is a critical concern in using immunoaffinity columns [9], [12], [30], [40], [41]. This setback is such that some authors have recently stated that increasing the number of antibodies from twelve to twenty has a limited beneficial impact, while significantly increasing the risk of removing peptides and proteins associated to the depleted proteins [12]. This risk is linked to the fact that, in non-denaturing conditions, the immunocaptured proteins that are known to function also as carriers remain associated with several peptides and proteins.

On the other hand, literature data have already shown a high degree of reproducibility of ProteoMiner beads, with a lower variability than other fractionation approaches, such as immunodepletion and gel filtration [26].

Finally, the workload and the cost to obtain the same protein quantity after fractionation by LAPs enrichment is significantly lower than performing the ProteoPrep20 immunodepletion (Table 3). Altogether, the ProteoMiner technology emerges as an attractive and convenient approach for plasma proteome analysis, especially as the first step of a complex orthogonal protocol.

**Table 3:** Comparison of costs and product characteristics.

Fractionation approach	n° of columns	Reusability	Plasma capacity per column	Protein recovery	Cost (€) per		Time (hours) required per	
					kit	1000 µl plasma	a single use	1000 µl plasma
ProteoMiner(large capacity kit)	10	Single use	1000 µl(∼70 mg of proteins)	∼3,7%(∼2.6 mg)	663	∼60	3	3
ProteoPrep20	1	100	8 µl(∼560 µg of proteins)	∼3%(∼17 µg)	1090	∼1300	0.5	62.5

## Supporting Information

Table S1 Proteins identified with at least 1 peptide and 99% confidence. The accession numbers, and the depletion/enrichment methods applied are also reported.
(XLS)

Table S2 All the peptides belonging to the proteins listed in Table S1 are reported in Table S2, together with their sequences, experimental masses, the difference between calculated and measured masses (expressed in ppm), possible variable modifications, ion scores, expectation values, ranking for Mascot searches, and XCorr, probability and ranking for Sequest searches.
(XLS)

Figure S1 **Polymeric contamination.** Examples of base peak chromatograms obtained after each step of the depletion protocol applied to an ultra-pure water sample. The polymeric contamination was observed after each step of the ProteoPrep20 depletion protocol applied to an ultra-pure water sample. Base Peak Chromatogram of: (A) a water sample; (B) water passed through the filter provided with the kit; (C) water passed through the depletion column; (D) water passed through the provided concentrator. (E) Example of the MS spectrum of contaminant species released into the sample.
(TIF)
