# Supplementary figures and images for: High Abundance Proteins Depletion vs Low Abundance Proteins Enrichment: Comparison of Methods to Reduce the Plasma Proteome Complexity
Source: PLoS One. 2011 May 4;6(5):e19603. doi: 10.1371/journal.pone.0019603 (PMC3087803; doi:10.1371/journal.pone.0019603)

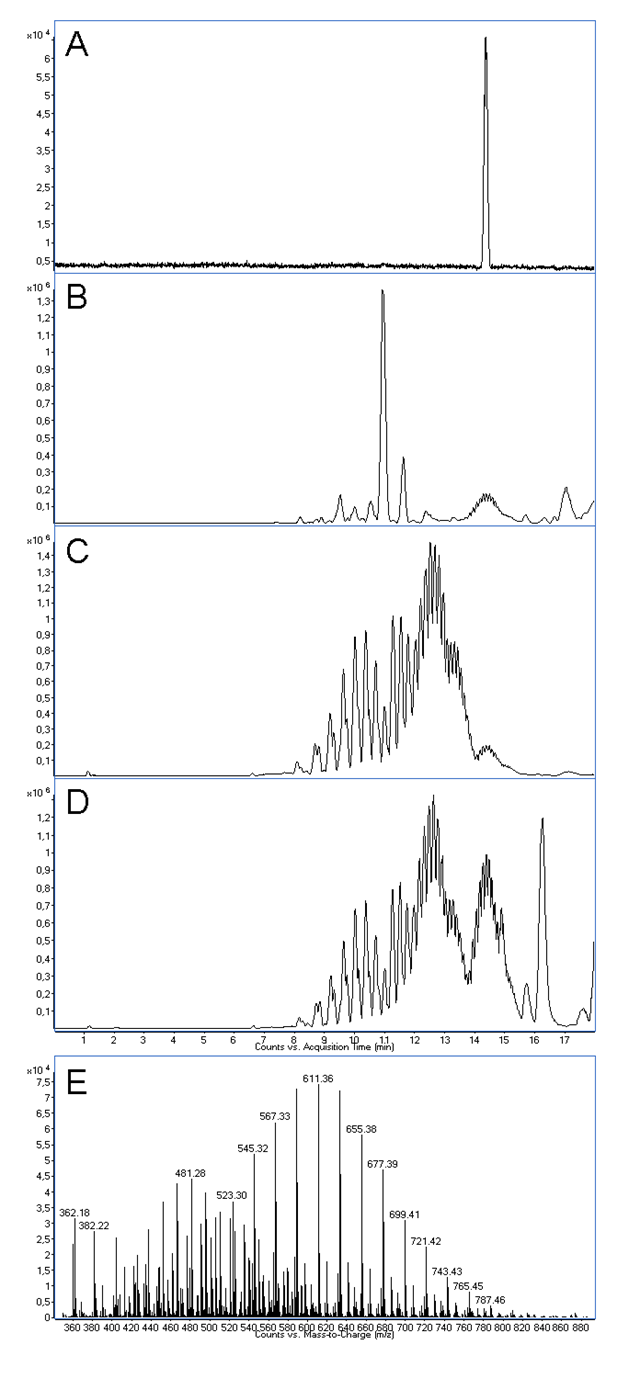

Supplement: Figure S1 — Polymeric contamination. Examples of base peak chromatograms obtained after each step of the depletion protocol applied to an ultra-pure water sample. The polymeric contamination was observed after each step of the ProteoPrep20 depletion protocol applied to an ultra-pure water sample. Base Peak Chromatogram of: (A) a water sample; (B) water passed through the filter provided with the kit; (C) water passed through the depletion column; (D) water passed through the provided concentrator. (E) Example of the MS spectrum of contaminant species released into the sample. (TIF) [file pone.0019603.s003.tif]
